# *Orientia tsutsugamushi* meningitis in a patient with tuberculous meningitis complications—a Case Report

**DOI:** 10.3389/fmed.2025.1591785

**Published:** 2025-06-25

**Authors:** Miao Tan, Hao Zou, MaiQing Yang, Anchun Liu

**Affiliations:** ^1^Department of Infection, Yueyang Central Hospital, Yueyang, Hunan, China; ^2^Department of Radiotherapy, Yueyang Hospital of Traditional Chinese Medicine, Yueyang, Hunan, China; ^3^Department of Pathology, Weifang People’s Hospital (First Affiliated Hospital of Shandong Second Medical University), Weifang, Shandong, China; ^4^Department of Pathology, Yueyang Central Hospital, Yueyang, Hunan, China

**Keywords:** scrub typhus, *Orientia tsutsugamushi* meningitis, tuberculous meningitis, next-generation sequencing, a rare case

## Abstract

Scrub typhus (ST) is caused by infection with *Orientia tsutsugamushi*, transmitted through chigger mite bites, resulting in a febrile disease. Clinical manifestations are complex and may be combined with other microbial infections. This report describes a rare case of scrub typhus meningitis and tuberculous meningitis. The patient presented with fever and chills, and also exhibited a dull reaction upon admission. Timely diagnosis and appropriate antibiotic therapy are critical for preventing complications and reducing mortality. Next-generation sequencing (NGS) provided diagnostic clues, and the diagnosis of scrub typhus meningitis combined with tuberculous meningitis was quickly clarified. The patient’s symptoms were relieved after appropriate treatment. NGS can be a valuable diagnostic tool for detecting early clinical infections.

## Introduction

Scrub typhus is a zoonotic acute febrile disease. It directly infects the endothelium of small blood vessels and causes systemic vasculitis (1), leading to clinical manifestations of multi-organ involvement, including meningitis and meningoencephalitis (2).

Tuberculous meningitis (TBM) is the most severe form of tuberculosis. The clinical manifestations of TBM are nonspecific during the early stages of the disease. Adults and children usually present with fever, headache, irritability, stiff neck, and vomiting. However, these symptoms make distinguishing TBM from other forms of meningitis difficult (3). Here, we report the rare case of meningitis caused by scrub typhus and tuberculosis.

## Case presentation

A 61-year-old male, who was engaged in masonry work, experienced a sudden onset of high fever and chills with a maximum temperature of 40°C on 8 October 2023, without any apparent cause. He had a dull reaction with slightly poor orientation and comprehension. Both pupils were equal in size and roundness, with sensitive light reflexes. Multiple lymph nodes in the neck and groin were enlarged, with a maximum size of approximately 2 × 1 cm, and had a medium texture. The white blood cell (WBC) count was 13.83 × 10^9^/L; neutrophils, 13.3 × 10^9^/L; neutrophil percentage, 96.2%; platelet count, 155 × 10^9^/L; and hemoglobin level, 127 g/L. Peripheral blood smears revealed some granulocytes with toxic morphological changes. Calcitoninogen (2.81 ng/L) and C-reactive protein (198.09 mg/L) levels were also determined. Based on these test results, we considered infectious fever and started doxycycline (0.1 g intravenous drops, q12 h) that day to fight the infection.

**Figure 1:**
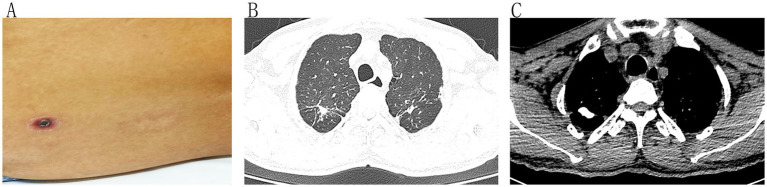
Clinical appearance and computed tomography. **(A)** The patient had a red scab on the back of his right lower back. **(B,C)** CT scans of the patient’s chest and secondary tuberculosis of both upper lungs (partial fibrosis and calcification).

Computed tomography (CT) of the head and chest revealed mildly ischemic cerebral white matter lesions. The results of the CT consider the possibility of secondary tuberculosis in both upper lungs (fibrosis and calcification). The patient’s family refused a lumbar puncture or magnetic resonance imaging (MRI) of the head. On 9 October, the patient’s impaired consciousness worsened, and he was manic. In addition, the patient was incontinent of urine. The following parameters were measured: albumin, 30.3 g/L; globulin, 26.6 g/L; total bilirubin, 34.63 μmol/L; direct bilirubin, 25.96 μmol/L; indirect bilirubin, 8.67 μmol/L; alanine aminotransferase, 231.34 U/L; aspartate aminotransferase, 334.32 U/L; glutamyl transpeptidase, 173.96 U/L; alkaline phosphatase, 277.3 U/L; adenosine deaminase, 99.99 U/L; and blood glucose, 3.86 mmol/L. *Orientia tsutsugamushi* (Supplementary Table S1) was detected using PMseq-RNA high-throughput metagenomic next-generation sequencing (mNGS) of pathogenic microorganisms (Huada Gene Technology Co. Ltd., Shenzhen, China) in the patient’s blood on 10 October (923 reads). After careful examination, the patient was found to have a 2 cm × 2 cm red crust on the right lumbar back (Figure 1A), confirming a diagnosis of scrub typhus.

With doxycycline, the patient remained recurrently hyperthermic with no improvement in central nervous system symptoms. CT of the patient’s lungs showed the possible secondary type of tuberculosis in both upper lungs with some fibrosis and calcification (Figures 1B,C). The family denied any history of tuberculosis or contact history, and a lumbar puncture was needed to exclude the possibility of combined TBM. Cerebrospinal fluid (CSF) intracranial pressure was 150 mm Hg, total cell count 116 × 10^6^/L, WBC count 97.00 × 10^6^/L, glucose 1.79 mmol/L, protein 2,492.6 mg/L (Supplementary Table S2). CSF general bacterial and fungal smears, new cryptococcal smears, antacid stains, and CSF cryptococcal pod antigen were all negative. T-cell test results for tuberculosis infection tuberculosis interferon gamma release assay (TB-IGRA) were negative. The pathological diagnosis (right cervical lymph node) was lymphoid tissue hyperplasia, mixed lymphocyte hyperplasia, and no apparent abnormalities were observed. Antibodies against hantavirus, wide reaction, and Weil–Felix reaction were negative. CSF was analyzed on October 11 for *Mycobacterium tuberculosis* by rifampicin resistance real-time fluorescence quantitative nucleic acid amplification test (GeneXpert MTB/RIF) and found to be negative for *M. tuberculosis* deoxyribonucleic acid (DNA) and rifampicin resistance. During this process, the patient still had recurrent high fever, impaired consciousness, and the highest temperature observed was 39.0°C. According to the results of the CSF suspected of TBM, methylprednisolone (40 mg, intravenous dose, once) was administered as a temporary measure to relieve systemic toxicity symptoms and reduce inflammation. We used glucocorticoids (GCs) in small doses for short periods to remain alert to potential tuberculosis (TB) infection, which can lead to systemic dissemination when GCs are used without anti-TB treatment.

**Figure 2:**
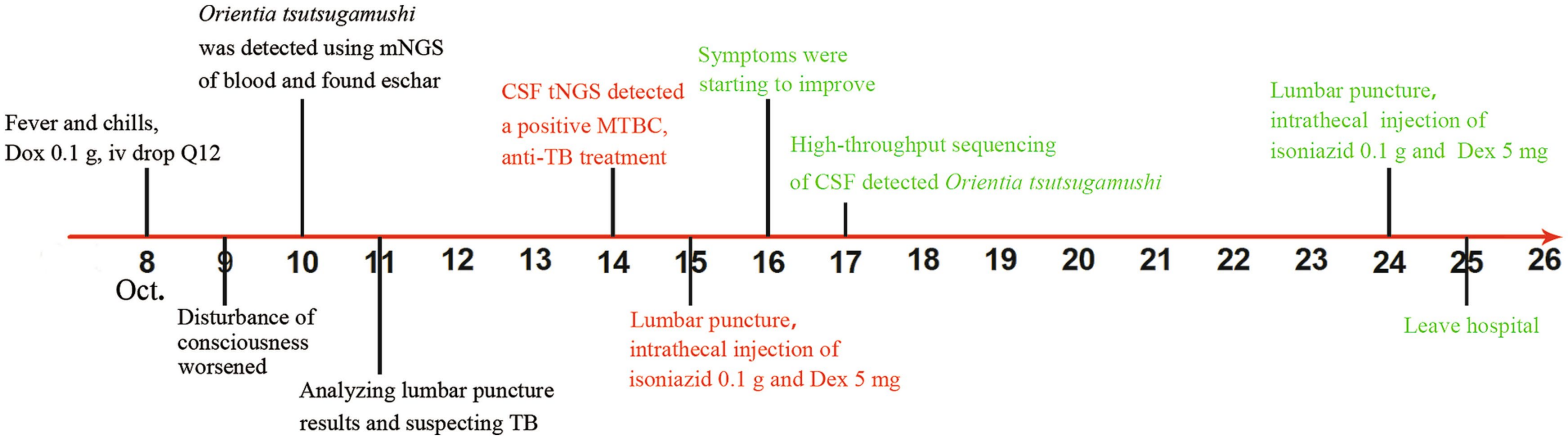
The timeline for the patient’s clinical course. Major events throughout the patient’s illness are highlighted in different colors on the line. Black indicates disease deterioration, red indicates the turning point, and green represents recovery. Dox, doxycycline; NGS, next-generation sequencing; CSF, cerebrospinal fluid; MTBC, *Mycobacterium tuberculosis* complex; TB, tuberculosis; Dex, dexamethasone.

On 14 October, CSF Tbseq for *M. tuberculosis*/non-tuberculosis mycobacteria identification and drug resistance genes targeted by high-throughput sequencing (tNGS) detected a positive *M. tuberculosis* complex group (2 reads) (Supplementary Table S1) (Shengting Medical Technology Co., Ltd., Hangzhou, China). Immediate anti-TB treatment was initiated. Isoniazid (0.4 g, intravenous drop, q.d.), rifampicin (0.6 g, intravenous drop, q.d.), moxifloxacin (0.4 g, intravenous drop, q.d.), and linezolid (0.6 g, intravenous drop, q.d.) were given in combination with anti-TB therapy. Dexamethasone sodium phosphate injection (15 mg, intravenous drops, q.d.) was administered to reduce inflammatory exudation and improve cerebrospinal fluid circulation, in addition to symptomatic combination therapy. The patient had abnormal liver function, and therefore, pyrazinamide was not administered during hospitalization. CSF bacterial and fungal cultures were negative. Multiple targeted amplification high-throughput sequencing of CSF detected *O. tsutsugamushi* (Supplementary Table S1) (Shengting Medical Technology Co., Ltd., Hangzhou, China), and a diagnosis of scrub typhus meningitis (STM) combined with TBM was precise (13 reads). The patient’s symptoms were relieved after appropriate treatment. The patient was released from the hospital on 25 October 2023. He continued to take oral anti-TB, hepatoprotective, and gastroprotective medications as well as prednisone acetate tablets (40 mg oral q.d.), which he gradually reduced to no more than 5 weeks later. On 5 December, the patient’s CSF was reexamined. Significant events throughout the patient’s illness are highlighted in different colors on the line (Figure 2). The Basic Local Alignment Search Tool, available on the National Center for Biotechnology Information website (NCBI)^1^, was used to examine nucleotide sequence similarity in public datasets. Sequence BLAST analysis revealed that the nucleotide similarity between the *M. tuberculosis* and *O. tsutsugamushi* sequences we obtained and the corresponding sequences available in GenBank were in the range of 92–95% and 89–98%, respectively. The nucleotide similarity of all sequences obtained in this investigation to the published sequences on NCBI exceeds 89%. Multiple sequence comparison results showed that the sequence similarity between *M. tuberculosis* and *O. tsutsugamushi* obtained in this study was less than 50%. The blood sample data generated in the present study are available in the NCBI database under the accession number PRJNA1268126 at https://www.ncbi.nlm.nih.gov/search/all/?term=PRJNA1268126. The CFS sample data generated in the present study are available in the NCBI database under the accession number PRJNA1267823 at https://www.ncbi.nlm.nih.gov/search/all/?term=PRJNA1267823.

## Discussion

Scrub typhus is an acute febrile illness caused by infection with *O. tsutsugamushi*, which is characterized by fever, rash, swollen lymph nodes, and eschar (4–6).

The patient presented with fever and chills, and also exhibited a dull reaction upon admission. mNGS can provide diagnostic clues, especially for infections that are difficult to recognize by traditional diagnostic methods (7). Blood mNGS results suggested scrub typhus, and after careful examination, an eschar 2 cm × 2 cm in size was found. The eschar was a special sign of scrub typhus (8). Based on clinical signs and mNGS test results, the diagnosis was scrub typhus. Liu X, et al. showed that the sensitivity of the mNGS test for scrub typhus was 100%, and the sensitivity of qPCR was 11.1% (4). Additional research indicated that mNGS could be a helpful diagnostic technique for determining the clinical microorganisms causing fever, especially those with atypical and severe symptoms (6, 9, 10).

Severe scrub typhus is associated with deadly consequences and significant complications. Common symptoms of organ involvement include acute respiratory distress syndrome, meningoencephalitis, and renal failure (11). Misra UK et al. concluded that up to two-thirds of patients with scrub typhus may develop meningoencephalitis or encephalopathy. Therefore, scrub typhus should be included in the differential diagnosis of febrile encephalopathy (12). In order to further clarify whether the central nervous system symptoms in this patient were caused by direct infection with pathogenic microorganisms, the patient’s CSF tNGS was further tested, and *O. tsutsugamushi* was detected. We diagnosed it as scrub typhus meningitis.

Cerebrospinal fluid features predictive of a diagnosis of STM include CSF WBC < 100 × 10^6^/L, CSF protein < 1,000 mg/L, and CSF sugar > 2.8 mmol/L (15). Another study shows that CSF examination revealed elevated protein (970 ± 483 mg/dL) in all patients (12). However, this is inconsistent with the patient’s CSF presentation; his CSF protein levels were very high (2,492.6 mg/L). With doxycycline, the patient continued to have recurrent high fever and no improvement in central nervous system symptoms. We considered that the patient’s lung CT showed tuberculosis foci with some fibrosis and calcification. Therefore, the possibility of combined TBM could not be ruled out. The clinical presentation of TBM is non-specific, especially in the early stages of the disease, when it is typically characterized by fever, headache, irritability, nuchal rigidity, and vomiting, making it difficult to distinguish it from other forms of meningitis (3). However, STM differs from TBM in terms of treatment options. If TBM is missed, it will inevitably lead to the patient’s death. Pathogenetic evidence of tuberculosis infection needs to be found as soon as possible. Multiple CSF samples failed to detect bacilli; CSF rapid *M. tuberculosis* cultures were negative, TB-IGRA was negative, and CSF GeneXpert MTB/RIF was negative. Finally, CSF tNGS suggested the presence of a positive *M. tuberculosis* complex. Although positive CSF antacid staining and *M. tuberculosis* cultures are the “gold standard” for diagnosing TBM, the low rate of positive antacid staining and the lengthy duration of *M. tuberculosis* cultures make both unsuitable for early TBM diagnosis (13). It has been suggested that mNGS represents an alternative method for detecting the presence of mycobacterial DNA in CSF samples from patients with TBM and is worth considering as a first-line CSF test (14). With appropriate anti-TB treatment, the patient’s symptoms were relieved, which indicated that the diagnosis was correct. In conclusion, multiple organ damage caused by severe infections should be diagnosed as early as possible, and the new generation of molecular testing techniques should be actively utilized, which was a crucial factor in achieving a successful treatment outcome in this case.

The study had limitations, as it was based on a single sample, and more samples are needed to determine whether the NGS test and diagnostic procedures used in the study are applicable.

## Data Availability

The blood sample data generated in the present study are available in the NCBI database under the following accession numbers PRJNA1268126 or at the following URL: https://www.ncbi.nlm.nih.gov/search/all/?term=PRJNA1268126. The CFS sample data generated in the present study are available in the NCBI database under the following accession numbers PRJNA1267823 or at the following URL: https://www.ncbi.nlm.nih.gov/search/all/?term=PRJNA1267823.

## Footnotes

^1^ www.ncbi.nlm.nih.gov/blast/
