# Supplementary material for: Orientia tsutsugamushi meningitis in a patient with tuberculous meningitis complications—a Case Report
Source: Front Med (Lausanne). 2025 Jun 25;12:1591785. doi: 10.3389/fmed.2025.1591785 (PMC12237661; doi:10.3389/fmed.2025.1591785)

Further confirmation of the obtained information was conducted through online BLASTn analysis.

The sequence details and BLASTn results obtained in this study are as follows:


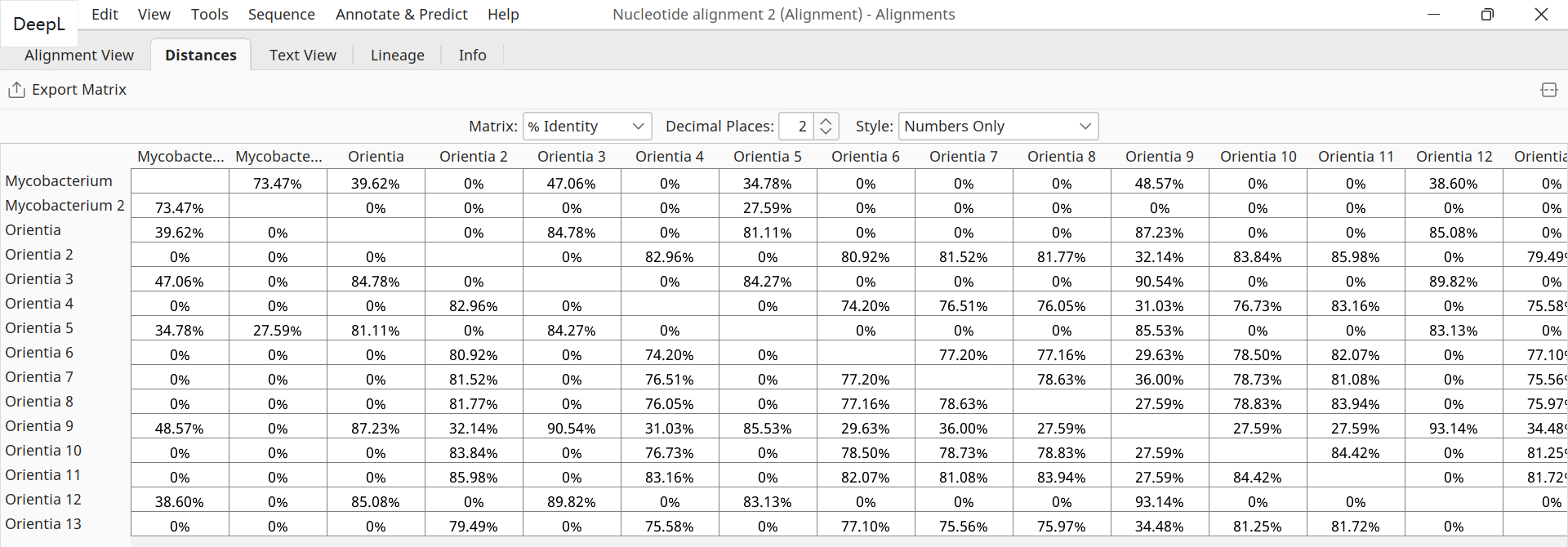


>Mycobacterium tuberculosis 1

GATGGACACACCGAAGGCATTGCCGAGTTTGATCAGCTCGGTCTTGTATAGGCCGTTGATCGTCTCGGCTGGTCATTGTCATAGGAGCTTCCGACCGCTCCGACCGACGGTGGATGCCTGCCTCGGCGAGCCGCTCGCTGAACCAGCGGATCGATGTTCATGAGATCCCTATCCGTATGGTGGATAACGTCTTTCAGGTCGAGTACGCCTTCTTGTTGGCGGGTCCAGATGGCTTGCTCTGATCGACGTCAGGACCGTAGGTGGCCATCGTGGAAGCGACCCGCCAGCCCAGGATCCTGCGAGCGTAGGCGTCGGTGACAAAGGCCGTAGGCGAACCTGCCCAGGTCGACACATGGAAGTGAGGTCTGCCCCCAGCCGGTTGGTGCTGGTGAGGGCCGAAGCGGCGCTGGACGAGATCGGCGGGACGGGCTGTGGCTTCGGATCAGCGATCGTGGTCCCCTGTGAGCTTCTGCCGGTGAGTCCCGGACAGGCGGTTTGGTCATCAGCCGTTCGACGGTGCATCTGGCCACCTCGATGCCCTCACGGTTCAGGGTTAGCCACACTGATGAGGCGACGAGCCGTGGATTAAGAGAATATGCTATCCTATGTTCT


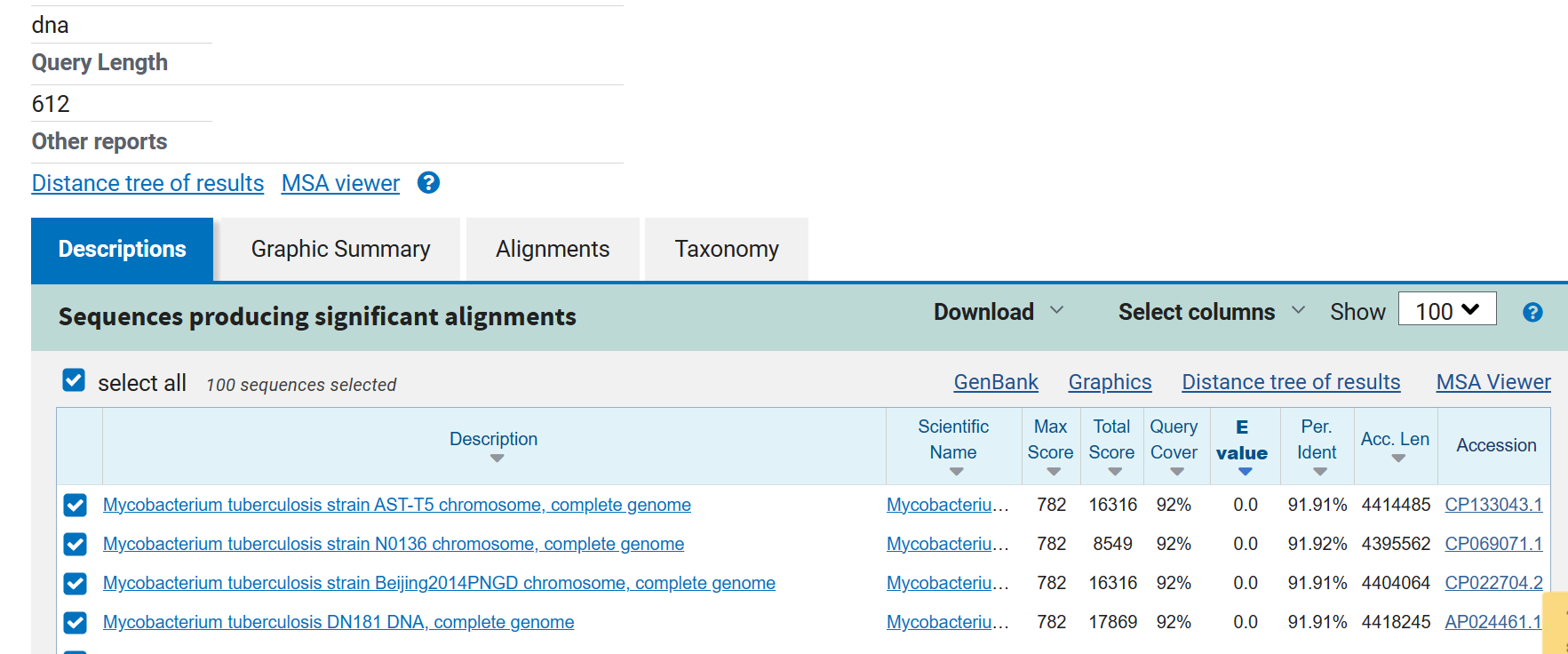


>Mycobacterium tuberculosis 2

GTATTGCTGGTGCTGCGGACAGACCTGGAATAGCGATTCTTAACGCCACCCATTGGTGCTGATGTTGCGTGGCCAACTCGACATCCTCATGGACCGCCAGGAGCTTGCCGGGTTTGATCAGCTCGGTCTTGTATAGGCCGTTGATCGTCTCGGCTAGTGCGTGTCATAGGAGCTTCCGACCGCTCCGACCGACGGTTGGATGCCTGCCTCGGCGAGCCGCTCGCTGAACCGGATCGATGTGTACTGAGATCCCTATCCGTATGGTGGATAACGTCTTTCAGGTCGAGTACGCCTTCTTGTTGGCGGGTCAGATGGCTTGCTCGATCGCGTCGAGACCATGGAGGTGGCCATCGTGGAAGCGACCCGCCAGCCCGGGATCACTTGCGAGCGTAGGCGTCGGTGACCAAAGGCCACATGTGACGGAAACCCTGCCCAGGTCGACACATAGGTGAGGTCTGCTACCCACAGCCGGTTAGGTGCGTGCTGGTGGTCGAAGCGGCGCTGGACGAGATCGGCGGACGGGCTGTGGCCGGATCAGCGATCGTGGTCCTGCAGGCTTTGCCGCGGGTGGTCCCGGACAGGCCGAGTTTGGTCATCAGCCGTTCGACGGTGCATCTGGCCACCTCGATGCCCTCACGGTTCAGGTTGCCACACT


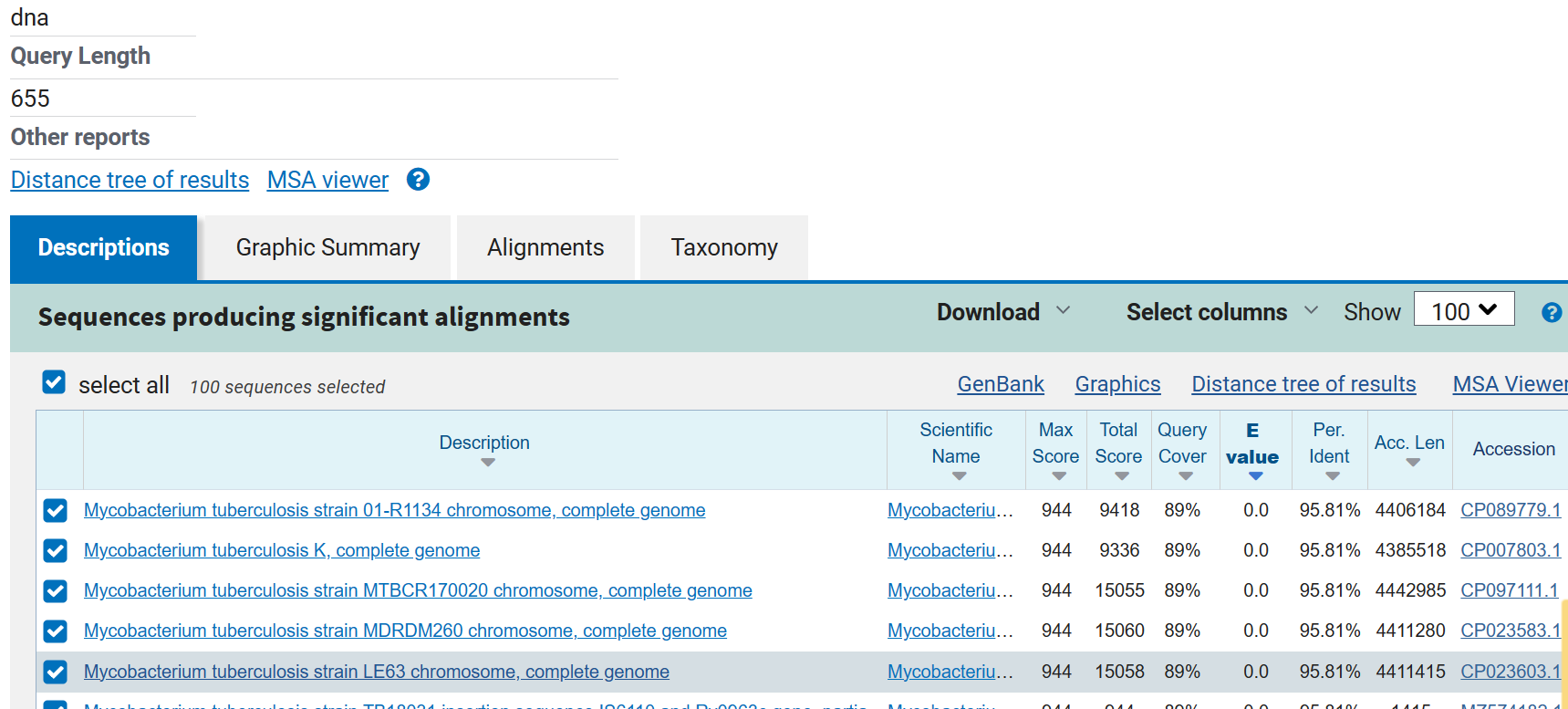


>Orientia tsutsugamushi 1

GACTCCGGGAGGCAGCAGTGGGGAATATTGGACAATGGGCGAAAGCCTGATCCAGCAATGCCGCGTGAGTGATGAAGGCCTTAGGGTTGTAAAGTAACTTTTAGTAGGGATGATAATGACAGTACCTACAGAAAAAGCTGACTAACTCCGTGCCAGCAGCCGCTGATGGCAGACGGAGGAGCTGGCGTTGTTCCGGAATTACTGGGCGTAAAGGAGCGCGTAGGCGGTTTAATAAGTTAGGAGTGAAATCCCGGGGCTTAACCCTGGAACTGGCACCCAAAACTGTTAGACTAGAGTATGGTGAGGATGATGGAATTTCTGGTGTGAAGGTAAAATTCTTAGATATTAGAAGGAACACCAGTGGCGAAAGCTGTCATCTGGACCATTACTGACGCTGAGGCGCGAAAGCGTGGGGAGCAAACAGGATTAGATACCCTGGTAGTCACGCCGTAAACGATGAGTGCTAGTTGTCGGCATGCATGCATGTCGGTGACGCAACTAACGCATTAAGCACTCCGCCTGGAGAGTACGGTCGCAAGATTAAAACTCAAAGAGAATTGACGGGGACCCGCA


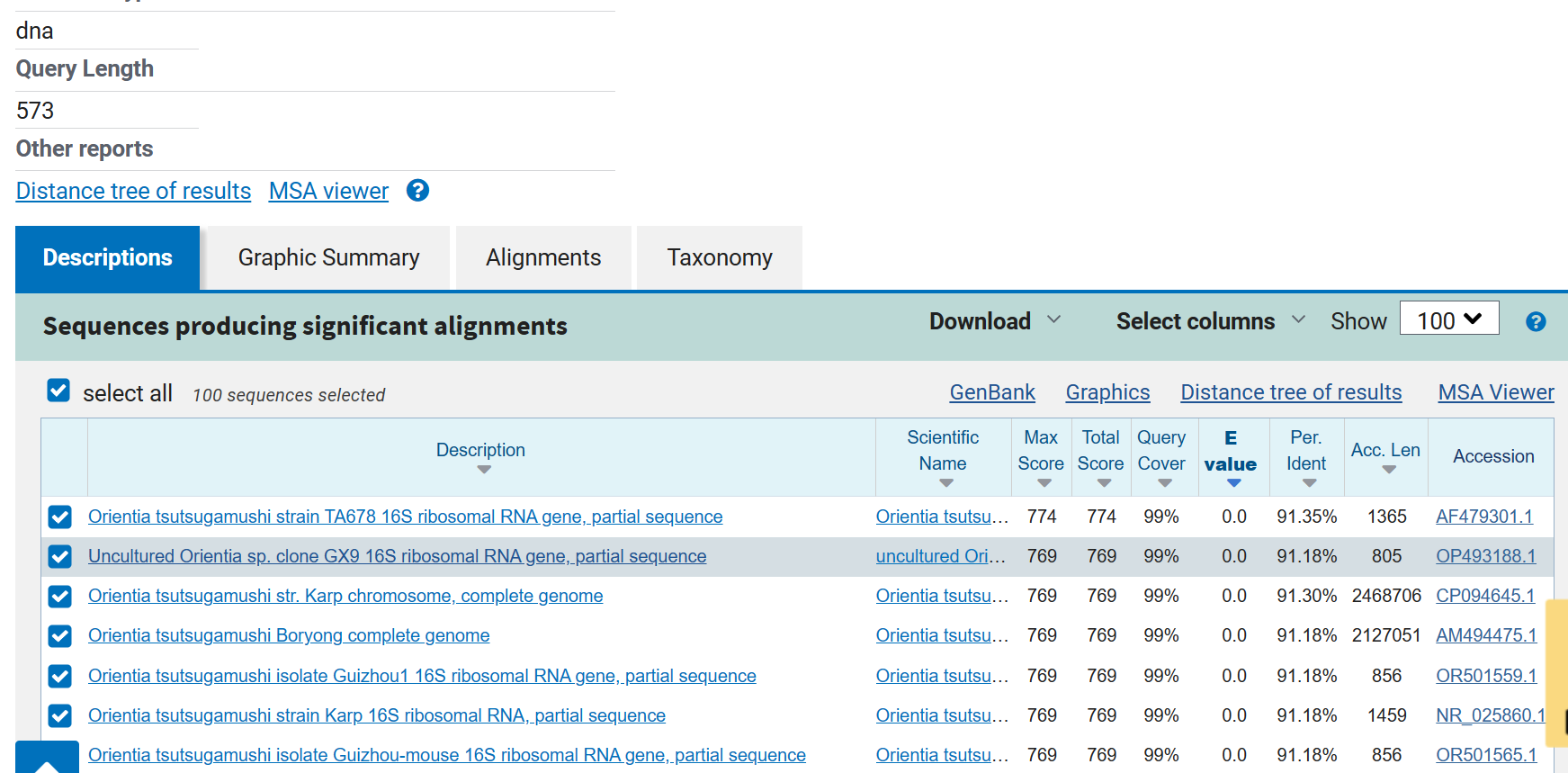


>Orientia tsutsugamushi 2

TTTGATTTTAATCTTGCGACCGTACTCCCCAGGCGGAGTGCTTAATGCTGATTAGCTACGAAACTGAAAAGAAAAATCCTCAATATCTAGCACTCATCGTTTACAGCGTGGACTACCAGGGTATCTAATCCTGTTTGCTCCCCACGCTTTCGCCTCAGCGTCAGTAATGGTCCAGATGACAGCTTTCGCCATAATTGTTCCTTCTAATATCTAAGGGTTACCTCTACACTAGAAATTCCATCATCCTCTACCATACTCTAGTCTAACAGTTTTAAAAGCAGTTCCAGGAGGTTAAGCCCTGGGATTTCCTCCTAACTTATTAAACCGCCTACGCGCCCGCGCCGCCAGTAGTAATTCCGAACAACGCCTAGCCCCCTCCGTCTTACCGCGGCTGCTGGCTGAGTTAGCCGGGGCTTTTTCTGTAGGTACTGTCATTATCATCCCTACTAAAGAAGCTTTAGCCCTAAGGCCTTCATCACTCGCGGCATTGCTGGATCGAGCTTTCATACGTTCTCCAATATTCCCACTGCTGCCTCCCGTAGGAGTCTGGGCCGTATCTCAGTCCCA


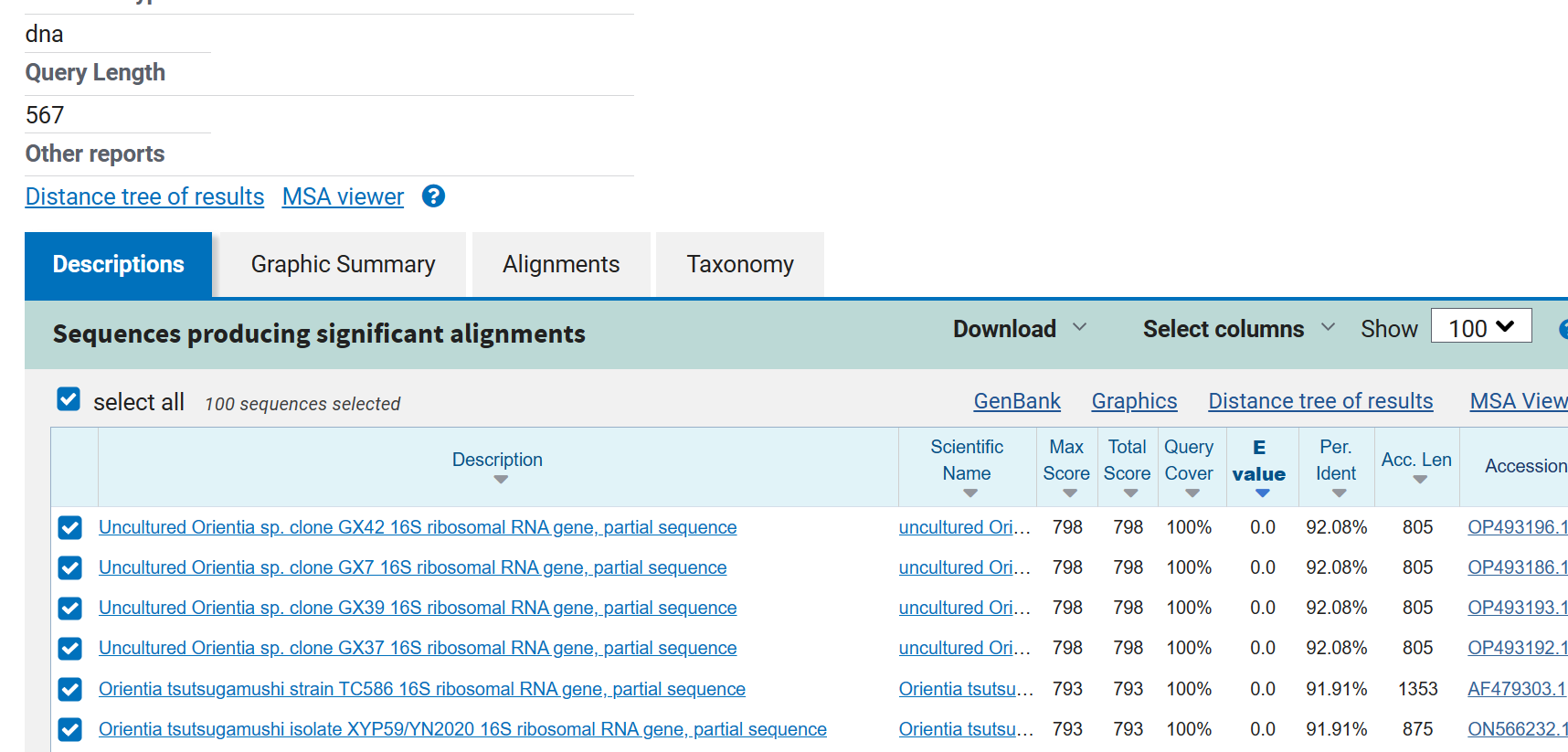


>Orientia tsutsugamushi 3

GGGAATATTGGACAATGAGGCGAAAGCCTGATCCAGCAATGCCGCGTGAGTAGTGAAGGCCTTAGGGTTGTAAAGCTCTTTTAGTAGGGATGATAATGACAGTACCTACAGAAAAAATACAAGCTAACTCCGTGCCAACAGCCGCGGTAAGACGGAGGGGCTAGCGTTGTTCGGAATTACTGGGCGTAAGGGCGCGTAGGCGGTTTAATAAGTTAGAATTAGAAATCCCAGGGCTTAACCCTGGAACTGCTTTAAAACTGTTAGACTAGAGTATGGTAGGGGATGATGGAATTTCTAGTGTAGAGGTAAAATTCTTAGATATTAGAAGGAACACCAGTGGCGAAAGCGTTTGTCATCTGGACCATTACTGACTGAGGCGCGAAAGCGTGGGGAGCAAACAGGATTAGGTACCCTGGTAGTCCACGCTGTAAACGATGAGTGCTAGATATTGGAGGATTTTTCTTTCAGTTTCATCAGCTAACGCATTAGCACTCCGCTAAGTGCGGTCGCAAGATTAAAACTCAAAGGAATTGACAGGGACCCGCA


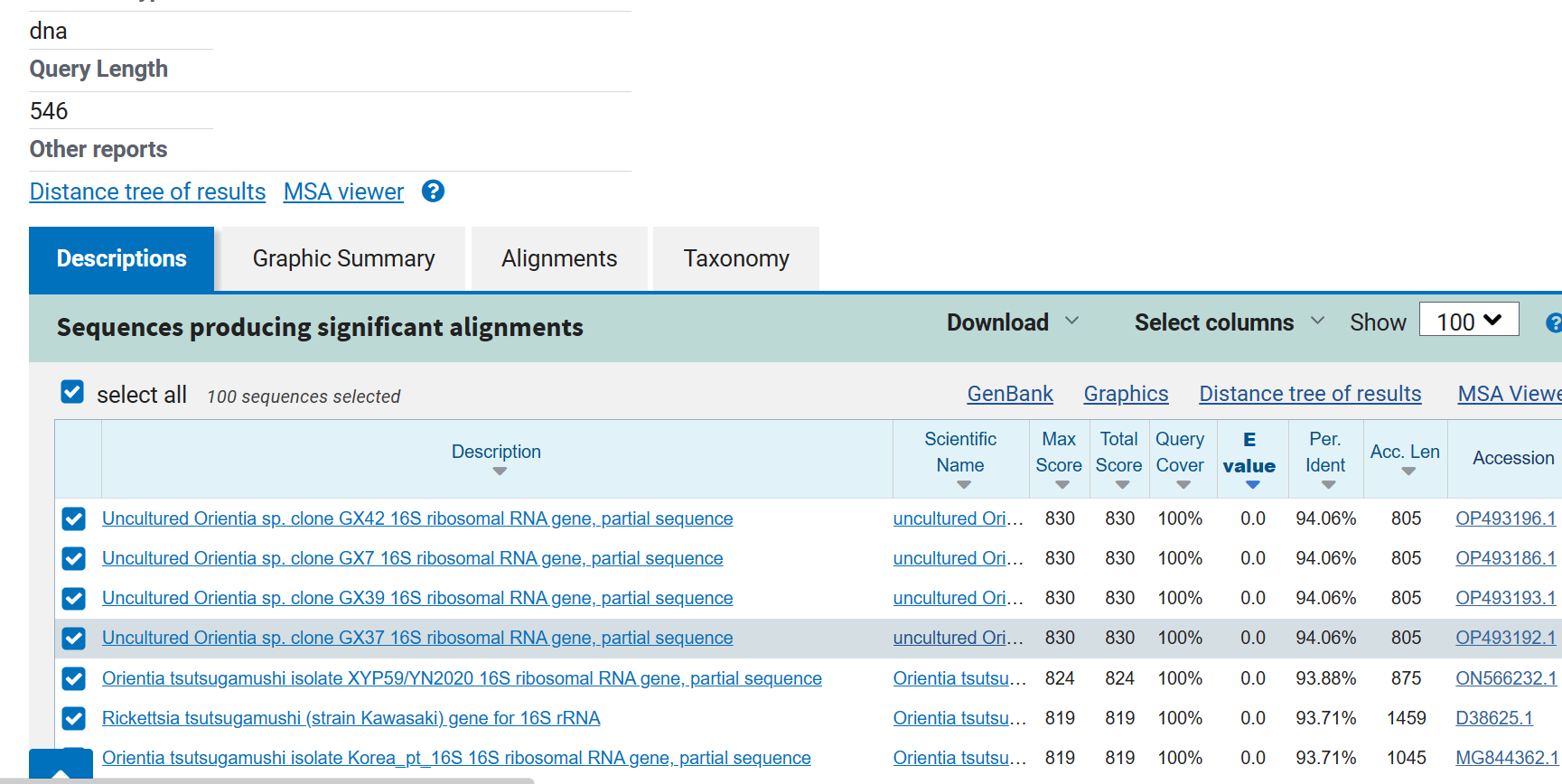


>Orientia tsutsugamushi 4

CTTTAGTTATAATCTTTACGACCGTACTCCCCAGGCGGAGTGCTAATGCGTTAGCTACAAGCTGGCAATCCTCAATATCTAGCACTCATCGTTTACAGCGTGGACTACCAGGGTATCTAATCCTGTTTGCTCCCCACGCTTTCGCGCCTCAGCGTCAGTAATGGTCCAGATGACAGCTTTCGCCACTGAGTGTTCCTTCTAATATCTAAGAATTTGCCTCTACACTGAAATTTCATCATCTCTGTACTCTAGTCTAACAGTTTTAAAAAGCAGTTCCAGGTTAAGCCCTGGGATTTCCTCTAATATTAAACTCGCCTGCGCGCCCTTTACGCCCAATAATGTGTTCGAACAGCGCTAGCCCCTCCGTCTTACCGCGGCTGCTGGCGCGAGGTGGCCGGGGCTTTTTCTGTAAAGTACTGTCATTATCATCCCTAAAAAGAGCTTTAGCCCTAAGGCCTTCATCACTCACGCGGCGTGCTGGATCAGGCTTTCGCCTTCATCCCAATATTCCCCACTGCTGCCCCCCGTAGGAGTCTGGGCCGTGTCTCAATGTCCCAGTGTGGCTGTTCGTCCTCTCAGACCAGCTGGATCACAGGCTTGGTAAGCCATTACCTTCAACTACCTAATCTGCCGCAGGCTCGTATCCATCAAGTAAATCTTTCCTCTTAGCAATGAGGCATACGGTATTAGCACTTATTTCTAAATGTTATTCCGTACCTGATGGGCAAATTCCCGATAGCTGTCACCGTTTTGCCACTAGTGTGCTAAGCAACTCAGCATTAATTCGTTCGACTGCATGTGTTAGGCATGCCGCCAGCGTTCGTTCTGAGCCAGAATCAGACTCTCCAC


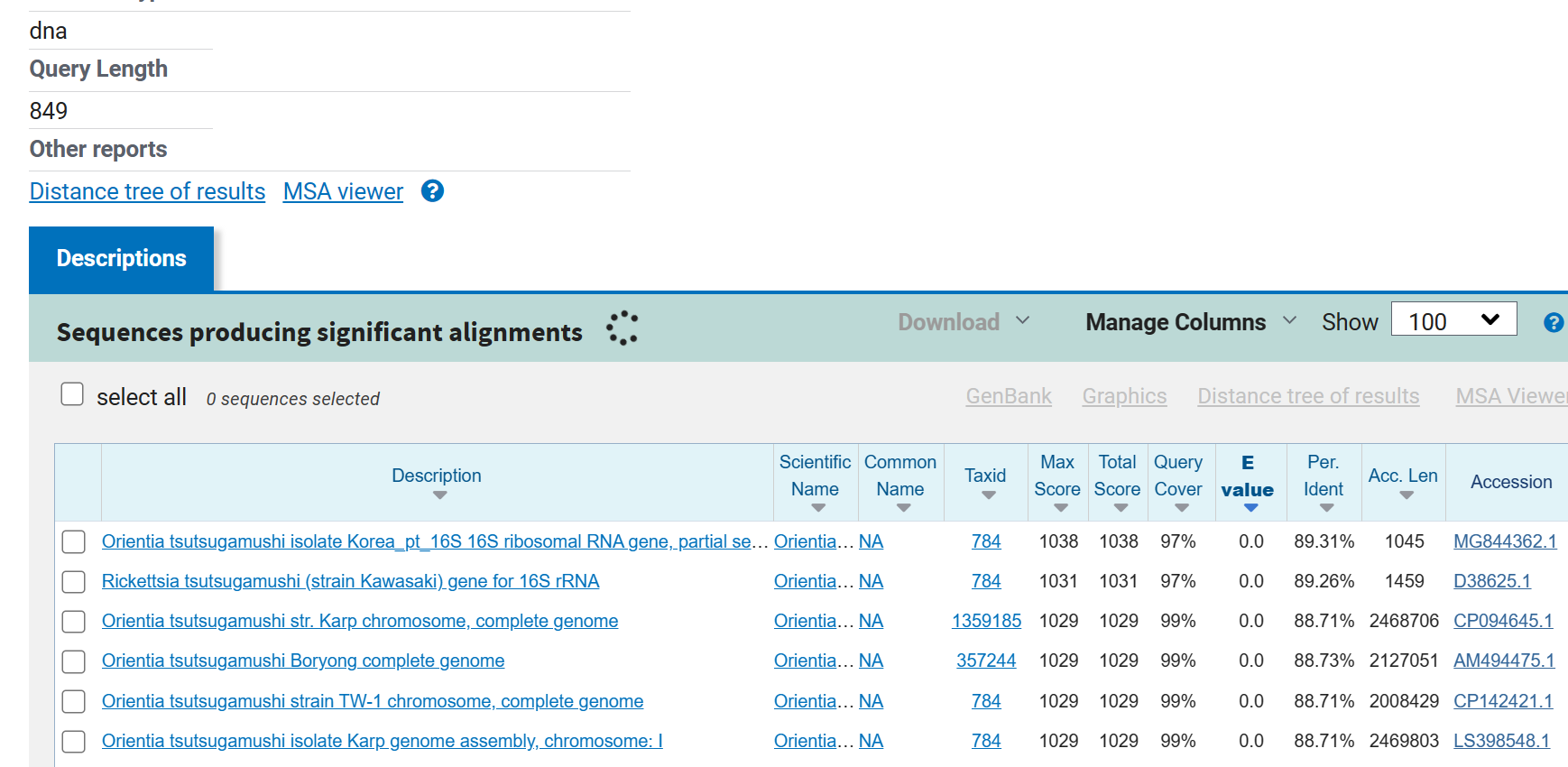


>Orientia tsutsugamushi 5

ATGCCTGGATGGTTCTGATCATCAATTAAAAAGCCGCCACCAATAGTTTGATTTTCGATACCTCCTACGAGGAGCAGCAGTGGGGGATATTGGACAATGGGCGAAAGCCTGATCCTTAATGCCGCGTGAGTGATGAAGGCCTTAGGGTTGTAAAGCTCTTTTAGTAGGGATGATAATGTCAGTACCTGCAGAAAAAGCCCAACTGAATCGTGCCAGCAGCCGCGGTAAGACGGAGGGAGCTGGCGTTGTTCCGGAATTACTGGGCGTAAAGGGCGCGTAGGCGGTTTAATAAGTTAGGAGTGAAATCCCCAGGGCCTAACCCTGGAACTGCTTTTAAAACATTAGACTGAAATTATGGTAGAGGATGATGGAATTTCTAGTGTAGAGGTAAAATTCTTAGATATTAGAAGGAACCACCGATGGCGAAAGCTGTCATCTGGACCATTACTGACTGAGGCGCGAAAGCGTGGGGGCAAACAGGATTAAGATACTGGTAGTCCACGCTGTAAACGATGAGTGCTGGGTGGAGGATTTTCTTTCAGTTTCGTAGCTAACGCGACTAAGCCTCCGCCTGGGAGTACGGTCGCAAGATTAAAACTCAAAGGAATTGACGGCGCA


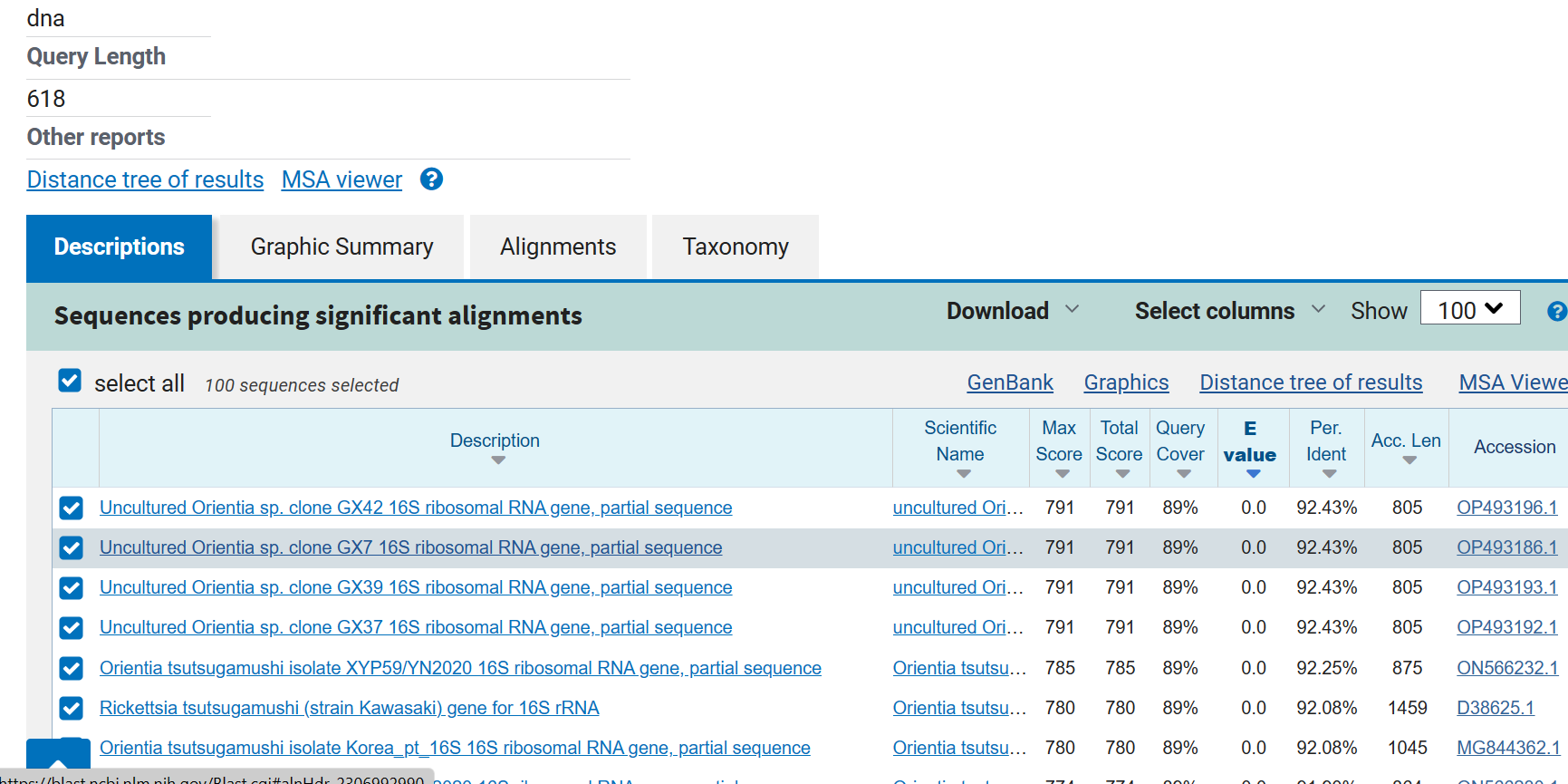


>Orientia tsutsugamushi 6

TGAGTTTTAATCTTGCGACCGTACTCCCCAGGCGAGTGTAATGCGTTAGCTTTACGAAACTGAAAGAAAAATCCTCCAATATCTAGCACTCATCGTTTACAGCGTGGACTACGGAGTATCCATCCTGTTTACTCCCCACGCTTTCACTTACAGCGTCGGTAATGGTCCAGATGACAGCTTTCGCCACTGGTGTTCCTTCTAATATCTAAGAATTTTACCTCTACACTAGAAATTCCATCATCCTCTACCTTCTCTAGTCTAACAGTTTTAAAAAGCAGTTCCAGGGTTAAGCCTGAAGGTTCCTCTAGCAATGAAGCCACCTACGCGCCCTTTACGCCCAGTAATTCCGAACAACACTAGCCCCCTCCGTCTTACCGCGGCTGCTGGCACGGAGTTAGCCGGGGCTTTTCTGTAGGTACTGTCGGTATCATCCCTGCTAAAAAGGCTTTACAACCTAAGGCCTTCATCACTCACGCGGCATTGCTGGATCGTTTCGCCCGGGTATCCCATTGTTCCTAACGCCCCGTAGGAGTCTGGAGCCGTATCTCAGTCCAGCAATATAAGCACCAACAGAAAGGTTAACGTAGATCAGGGTCT


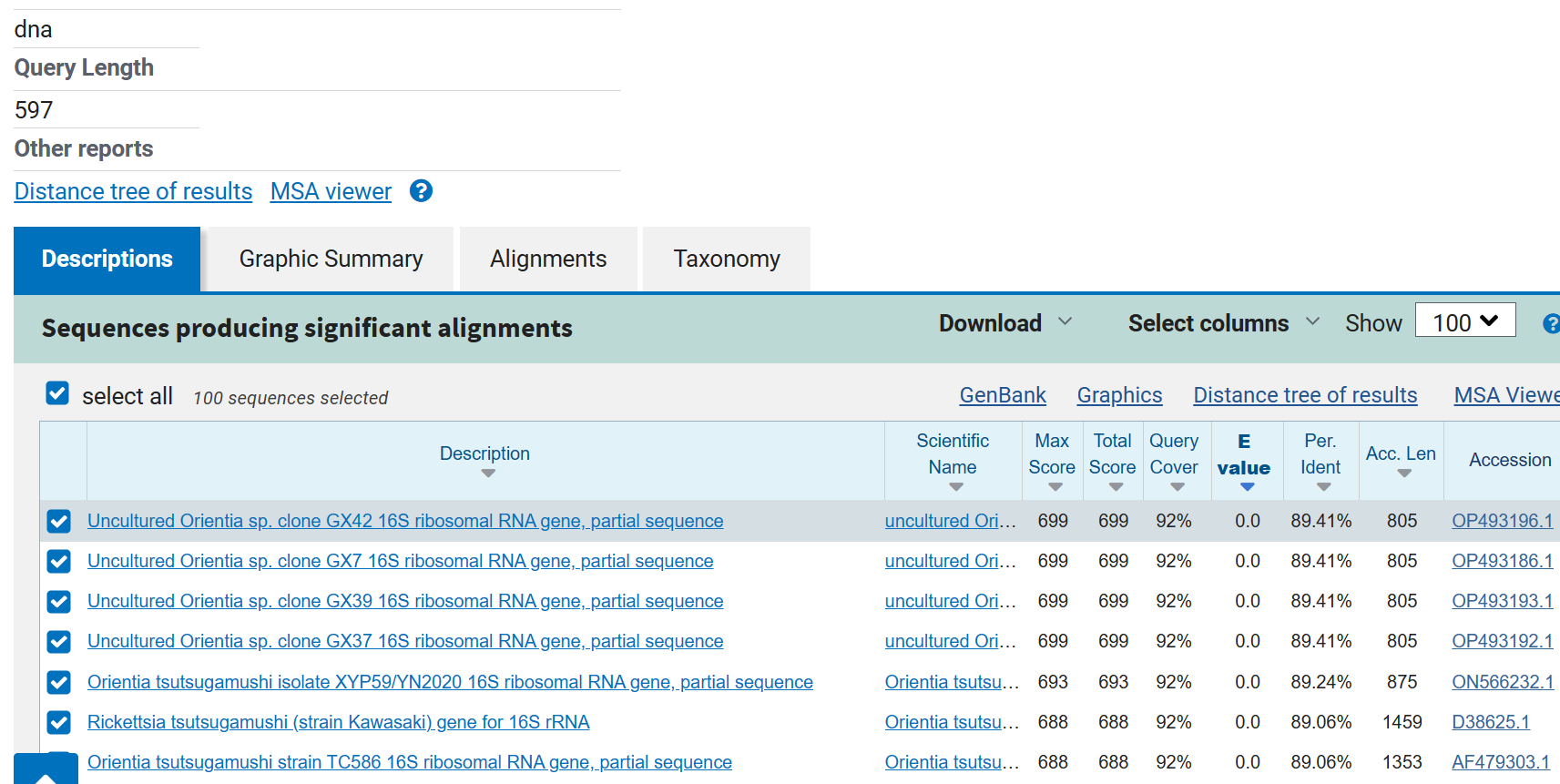


>Orientia tsutsugamushi 7

GTTTTTAATCTTCGCGACCATGCTCCCCAGGCCGGGTGTACGTTAGCTACGAAACTGAAAAAAATCCTCCCAATATCTAGCACTCATCGTTTACGACGTGGACTACGGGAGTATCTAATCCTGTTTATCACCTTTCGCGCCTCAACGTCAGTAATGGTCAGATGACAGCTTTCGCCTGGTGTTCCTTCTAATATCTAAATTTTACCTCTACACTAGAAATTCCATCATCCTCTACCATACTCTAGTCTAACGGTTTTAAAAGCAGTTCCAGGTTAAACCTAGGTTTCGCTCTAGCTTATTAAACCGCCTACACGCCCTTCCTTAGTAGTTCCGAACAACGCTAGCCCCTCCGTCTTACCGCGGCTACTGGCACGGAGTTAGCCAGGGCTTTCTGTAGGTACTGTCATTATCAGCCCTACTAAAGGCTTTACAACCTAAGGCCTTCATCACTCATGACATTGCTGGATCAGGCTTTCGCCCATTGTCCAATATTCCCCACTGCTGCCCCGTAGAGTCTGGGCCGTATCTCAGTCCCA


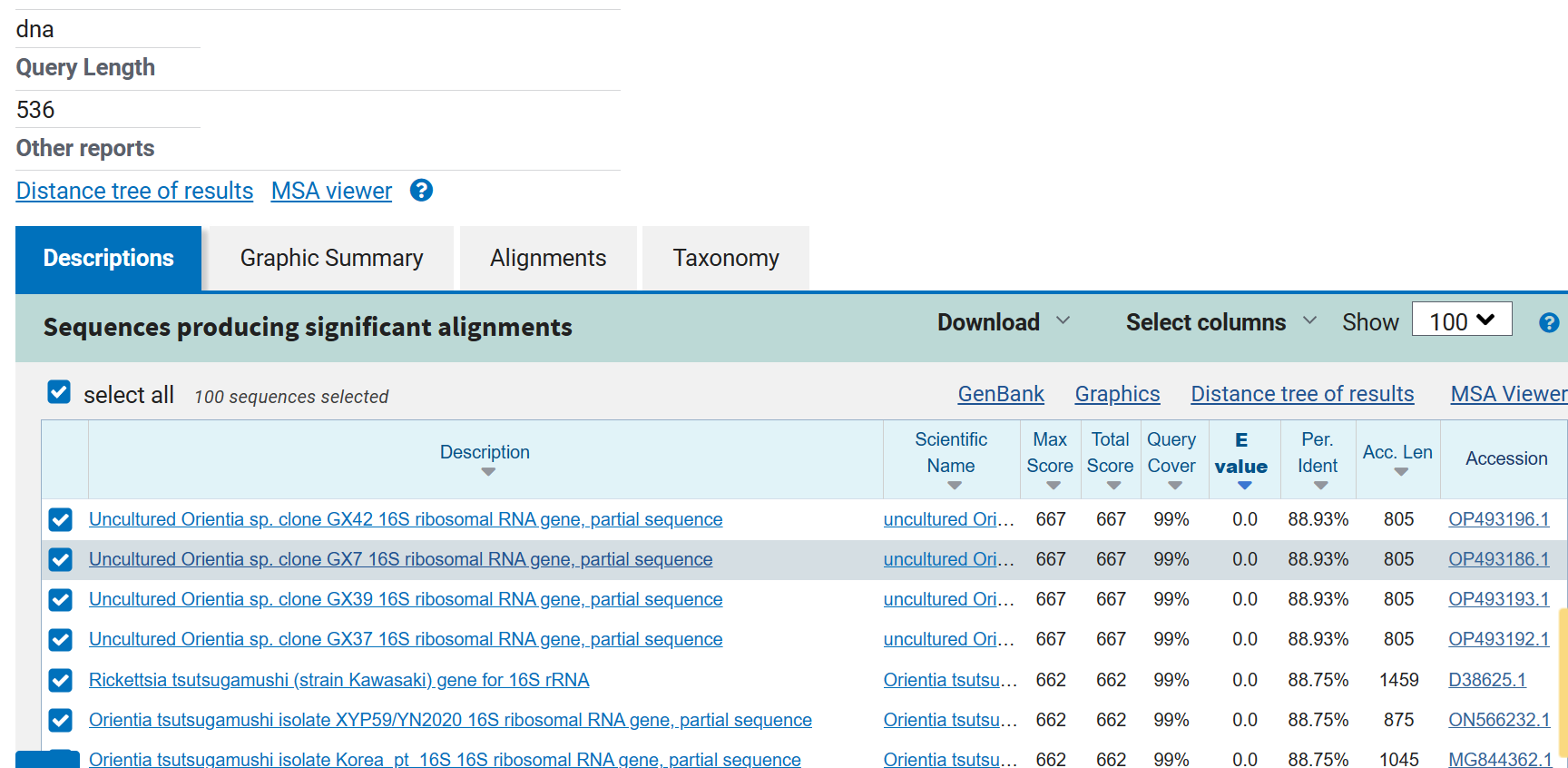


>Orientia tsutsugamushi 8

TTTGAGTTTTAATCTTGCGACCGTACTCCCCAGGCGGTGTGCTTAATGCGTTAGCTGCGTGCCGACATGCATGCATGCCGACAACTAGCACTCATCGTTTACGGCGTGGACTACCAGGAGGTATCTAATCCTGTTGCTCCCCACGCTTTCGCGCCTCAGCGTCAGTAATGGTCCAGATGACAGCTTTCGCCACTGGTGTTCCTTCTAATATCTAAGAATTTTACCTCTACACTAGAAATTCCATCATCCTCTGCCATACTCCAGTCTAACGTTTTCAAACAGTTCCAGGGTCAAGCCCTGGGATTTCCTCTAACTTATTAAACCGCCTACGCGCCCTTTACGCCCAGTAATTCCAGACAACGCTAGCCCTCCGTCTTACCGCGGCTGCTGGCACGGAGTTAGCCGGGGCTTTTTCTGGTACTGTCATTATCATCCTACTAAAAGGCTTTACACAACTAGGCCTTCATCACTCACGCGGCATTGCTGGATTGGTATCCCATTGTCCAATATTCCCTGCTGCCCCCGTAGGAGTCTGGGCCGTATCTCAGTCCCAGCAATATCAGCGCCAACAAGGAAAAGGTTAACGG


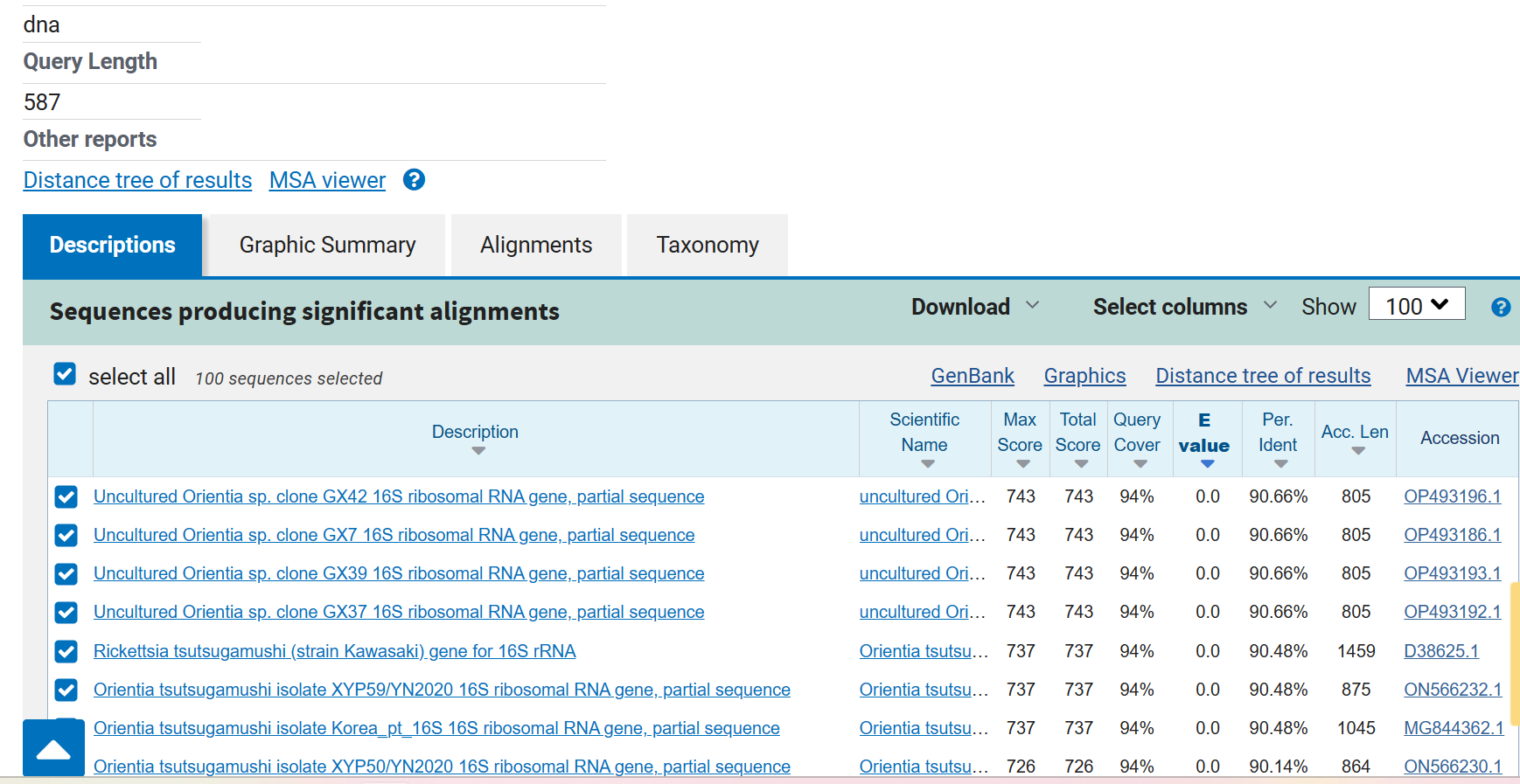


>Orientia tsutsugamushi 9

TGGGGAATATTGGACAATGGGCGAAAGCCTGATCAGCAATGCCGCGTGAGTGATGAAAGGCCTTAGGGTTGTAAAGCTCTTTTAGTAGGGATGATAATGACAGTACCTACAGAAAAAGCCCCGGCTAACTCCGTGCCAGCAACCGCGGTAAGACGGAGGGGCTAACGTTGTTCGGAATTACTGGGCGTAAAGGGCGCGTAGGCGGTTTAATAAGTTAGGAGTGAAATCCCAGGGCTTAACCCTGGAACTGCTTTTAAAACTGTTAGACTAGAGTATGGTAGAGGATGATGGAATTTCTAGTGTAGAGGTAAAATTCTTAGATATTAGAAGGAACACCAGCGGCGAAAGCTGTCATCTGGACCATTACTGACGCTGAGGCGCGAAGCGGGAGCAAACAGGATTAGATACCCTGGTAGTCCACGCTGTAAACGATGAGTGCTAGATATTGGAGGATTTTTCTTTCGGTTTCGTAGCTAACGCATTAAGCACTCCGCCTGGGGAGTACGGTCGCAAGATTAAAACTCAAAGGAATTGACGGGGACCCGCAGAAGATAGAGCGATGAGCAAGAGCAATACG


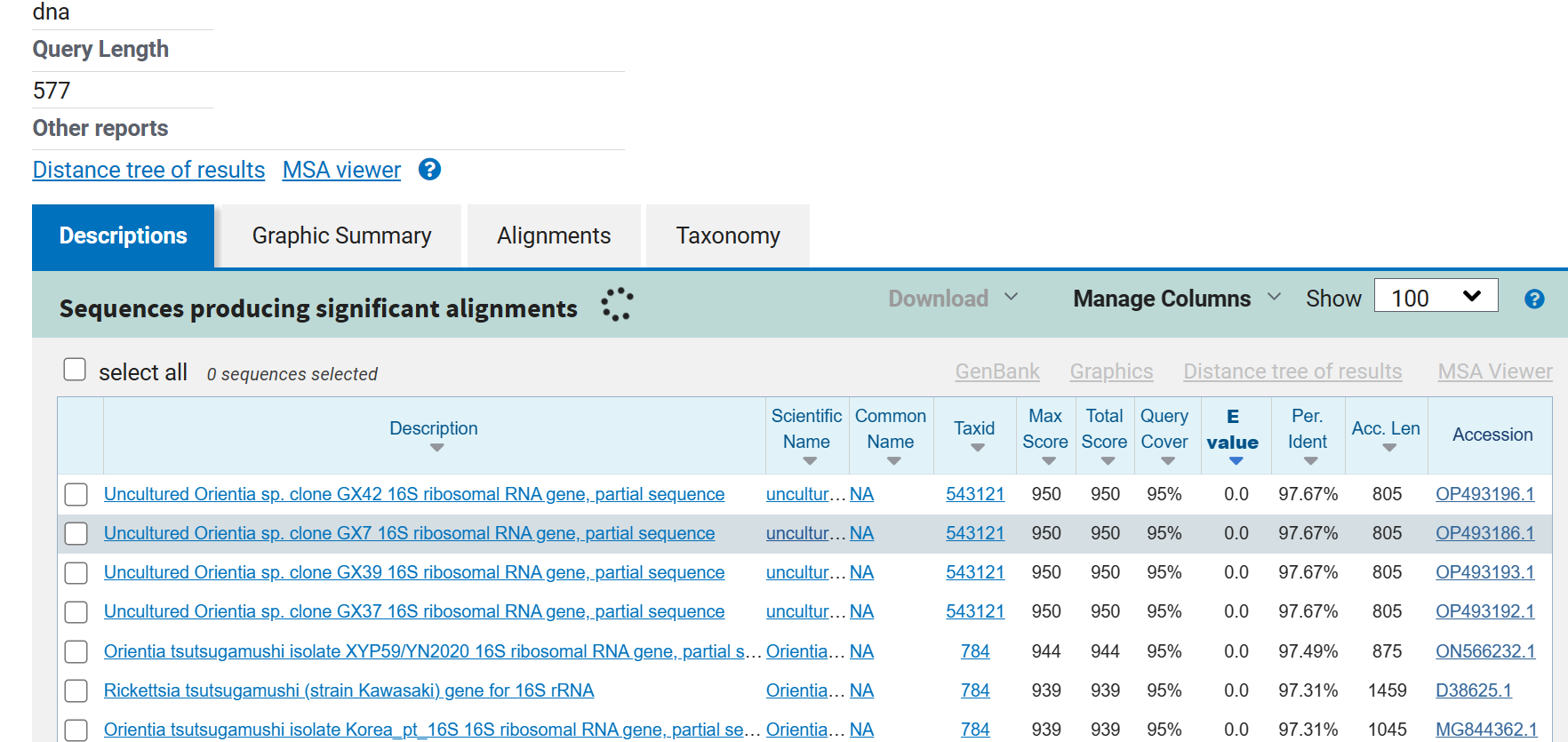


>Orientia tsutsugamushi 10

TTTGAGTTTTTATCTTGCGACCGTACTCCCCGGGCAGGTGCTTAATGCGTTAGCTACGAAACTGAAGGGAAAAATCCTCCAATATCTAGCACTCATCGTTTACAGCGTGGACTACCAGGGTATCTAATCCTGTTTGCTCCCCACGCTTTCGCTTACTCCAGCGTCAGTAATGGTCCAGATGACAGCTTTCGCCACTGGAGTTCCTTCTAATATCTAAGAATTTTACCTCTACACTAGAATTCCTCATCCTCTACATACTCTAGTCTAACAGTTTTAAAGAAAACGCAGTTCCAGGGTTAAGCCCTGGGATTTCACTCTAACTTATTAAACCGCCTACGCGCCCTTTACGCCCAGTAATTCCGAACAACTAGCCCCCTCCGTCTTACCGCGGCTGCTGGCACGGGTTAGCCGGGGCTTTTTCTGTAGGTACCGTCATTATCATCCCTACTAAAGAGCTTTACAACCCTAAGGCCTTCATCACTCACGCAGCATTGCCGGATCAGGCTCGCCCATTATCCAATATTCCCCGCTGCGGCCTCCCGTAGAAGTGCAATATCAGCACCAACAGAAGGTTAACGTAGATCAGGGTCTCT


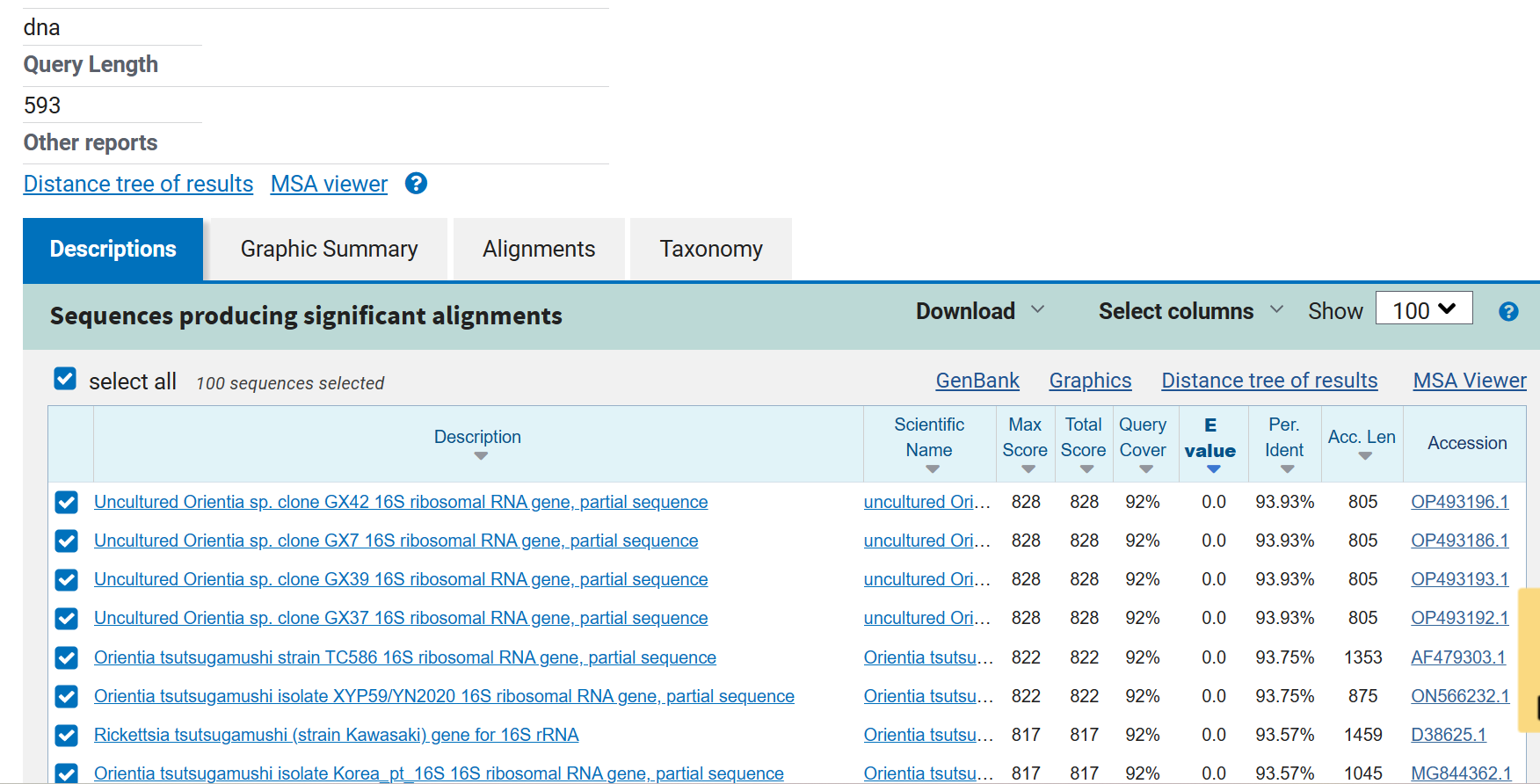


>Orientia tsutsugamushi 11

TTTGAGTTTTAATCTTGCGACCGTACTCCCCAGGCGGAGTGCTTAATGCGTTAGCCCTTGAAACTGAAGAAAAATCCTCCAATATCTAGCACTCATCGTTTACAGCGTGGACTACCAGGGTATCTAATCCTGTTTGCTCCCCACGCTTTCGCGCCTCAGCGTCAGTAATGGTCAGATGACAGCTTTCGCCACTGGTGTTCTTTCTAATATCTAAAAATTTTACCTCTACACTAGAAATTCCATCATCTCTCTGTACTCTAGTCTAACGTTTTAAAAGCAGTTCCAGGGTTAAGCCCTGGGATTCCTCCTAGCAGTAAACCGCCTACGCGCCCTTTACGTCCAGTAATTCGAACAACGCTAGCCCCTCCGTCTTACCGCGGCTGCTGGCACAGGTTAGCCGGGGCTTTTTCCGTAGGTACTGTCATTATCATCCCCTACTAAAAGAGCTTTAGCCCTAAGGCCTTCATCACTCACGCGGCATTGCTGGATCAGGCTTTCGCCCATTGTCCAATATTCCCCACTGCTGCCCCCGTAGGAGTCTGGGCGTGTCTCAGTCCCG


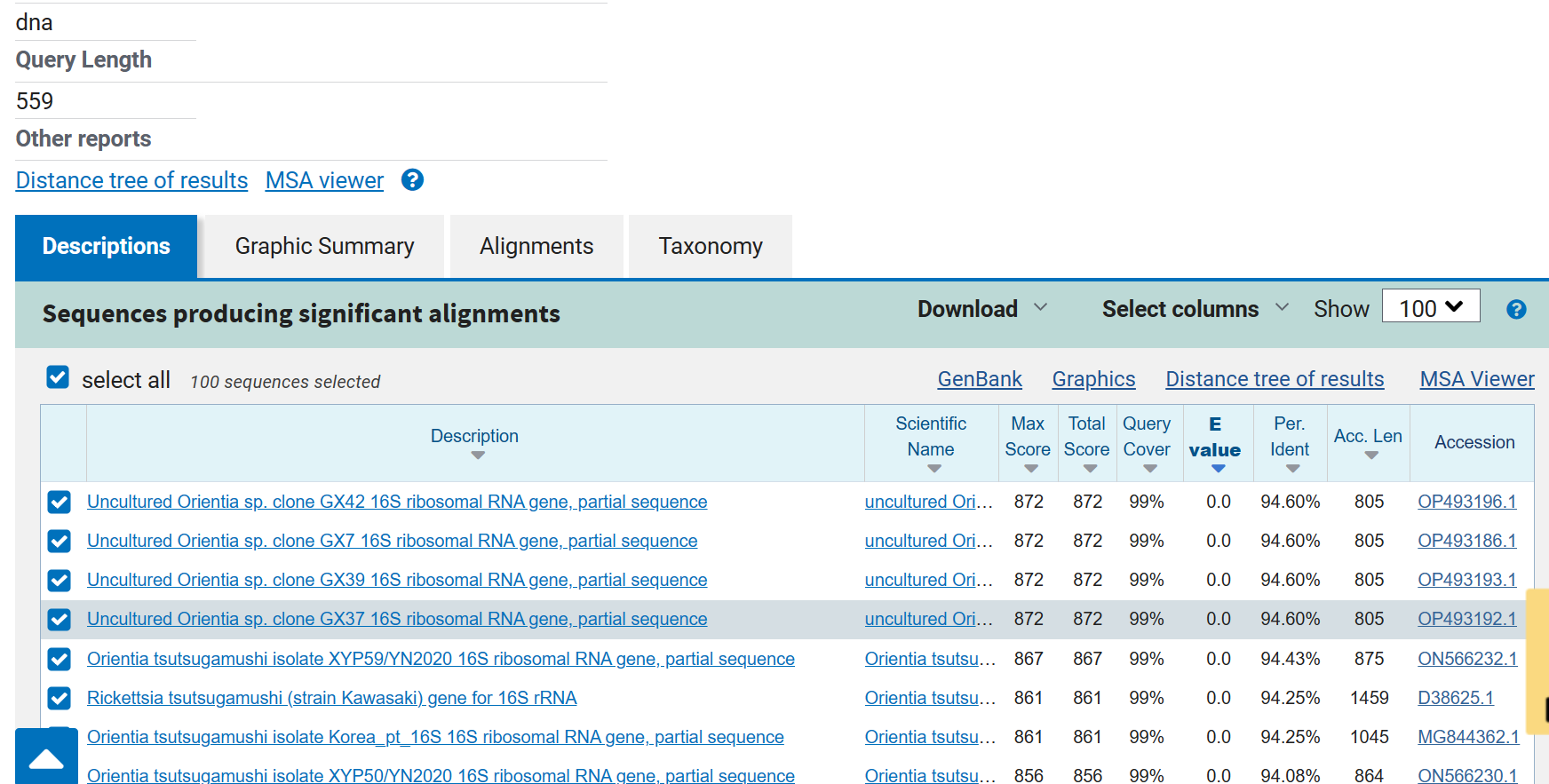


>Orientia tsutsugamushi 12

GACTCCTACAAGAGGGCAGCAGTGGGGAATATTGGACAATGGGCGAAAGCTGATCCAGCAATGCCGCGTGAGTGATGAAGGCCTTAGGGTTGTAAAGCTCTTTTAGTAGGGATGATAATGACAGTACCTACAGAAAAAGCCCAGTAACTCCGTACCAACAGCCATGATAAGCAAGAGGGGCTAGCGTTGTTCAGAATTACTGGGGCGTAAAGGGCGCGTAGGCGGTTTAATAAGTTAGGGGTAGAAATCCCAGGGGCTTAACCCTGGAACTGCTTTTAAAACTGTTAGACTAGAGTATGGTAGAGGATGATGGAATGTCTAGTGTAGTAAAATTCTTAGATATTAGAAGGAACACCAGTGGCGAAAGCTGTCATCTGGACCATTACTGACGCTGAGGCGCGAAAGCGTGGGAGCAAACAGGATTAGATACCCTGGTAGTCCACGCTGTAAACGATGAGTGCTAGATATTGGAGGATTTTTCTTTCAGTTTCGTAGCTAACGCATTAAGCACTCCGCCTGGGAGTACGGTCGCAAGATTAAAACTCAAAGGAATTGACGGGGACCCGCA


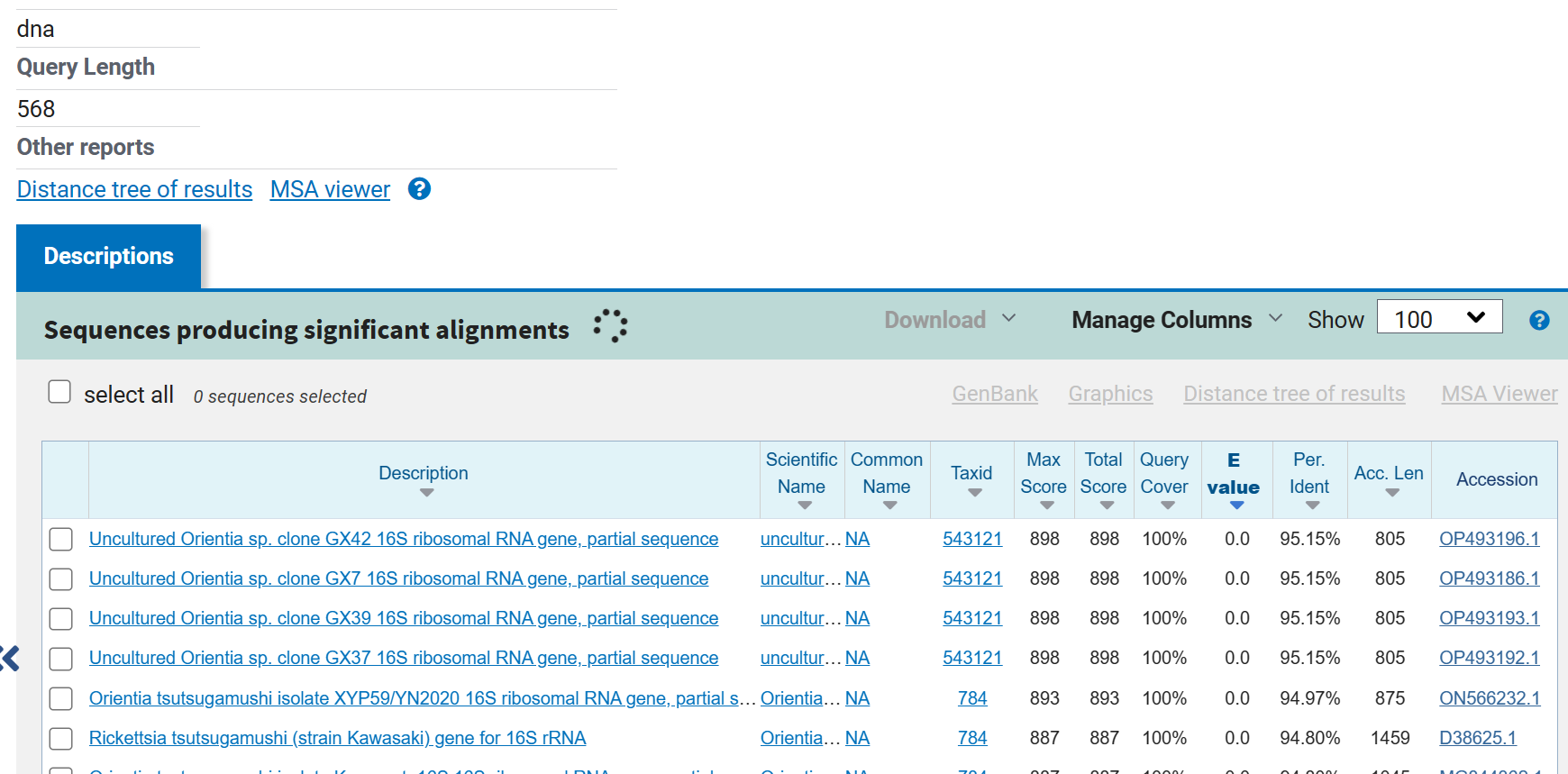


>Orientia tsutsugamushi 13

CTTTGAGTTTAATCTTGCGACCATGCTCCCAGGCGGAGTGTAATGCGTTCTTCTGAAACCTGAAAGAAAAATCCTCCAATATCTAGCACTCATCGTTTACAGCATTGGACTACCAGGGTATCTAATCCTGTTTGCTCCCCACGCTTTCGCGCCTCAGCGTCAGTAATGGTCAGATGACAGCTTTCGCCACTGGTGTTCCTTCTAATATCTAAAAATTTTGCCTCTACACTAAATTCCATCATCCTCTACCATACCCTAGTCTAACAGTTTTAAAAGCAGTTCCAGGGGTTAAGCGCAAGAGTTCCTCCTAACTTGATAAACTAACCTACGCGCCCTTTACGCCCAGTGATTCCGAGCAACATAGCCCCCTTCGTATTACCACCTTGCTGGCACGAAGTTAGCCAGGGCTTATTCTTACGGTACCGTCATTATCTTCCCGTACAAAAGAGCTTTACAACCCTAGGGCCTTCATCACTCACGCGGCATGGCTGGATCAGGCTTGCGCCCATTGTCCAATATTCCCCACTGCAGCCTCCCGCAGAAGTGCAATATCAGCACCAGCAAGAAAA


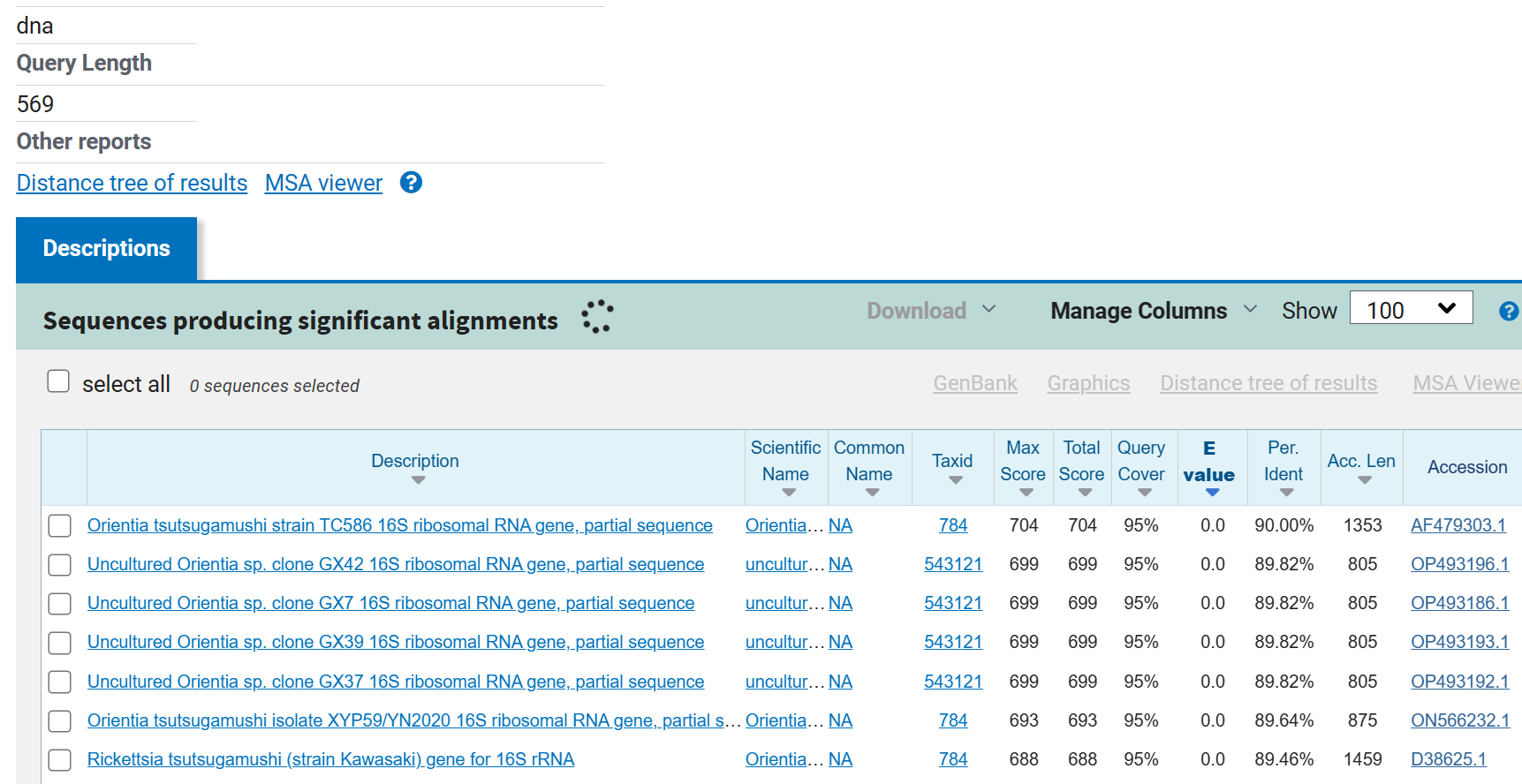

Supplement: Supplementary file 1 [file Data_Sheet_1.DOCX]
