# Supplementary material for: Orientia tsutsugamushi meningitis in a patient with tuberculous meningitis complications—a Case Report
Source: Front Med (Lausanne). 2025 Jun 25;12:1591785. doi: 10.3389/fmed.2025.1591785 (PMC12237661; doi:10.3389/fmed.2025.1591785)
Supplement: Supplementary file 2 [file Table_1.DOCX]

**Supple. Table 1 NGS pathogens detection results.**

| Sample | Pathogens detection | Detection Methods | Reads |
| --- | --- | --- | --- |
| Blood | *Orientia tsutsugamushi* | PMseq-RNA high-throughput metagenomic next-generation sequencing | 923 |
| CSF | Mycobacterium tuberculosis complex group | Nanopore-based targeted next-generation sequencing | 2 |
| CSF | *Orientia tsutsugamushi* | multiple targeted amplification-high-throughput next-generation sequencing | 13 |
