# Supplementary material for: Orientia tsutsugamushi meningitis in a patient with tuberculous meningitis complications—a Case Report
Source: Front Med (Lausanne). 2025 Jun 25;12:1591785. doi: 10.3389/fmed.2025.1591785 (PMC12237661; doi:10.3389/fmed.2025.1591785)
Supplement: Supplementary file 3 [file Table_2.DOCX]

**Supple. Table 2 Results of laboratory tests of the patient’s CSF on different days.**

|  | Reference Value | Day1  (8th Oct) | Day 3  (10th Oct) | Day 4  (11th Oct) | Day 5  (12th Oct) | Day 8  (15th Oct) | Day 17  (24th Oct) | Fellow-up  (5th Dec) |
| --- | --- | --- | --- | --- | --- | --- | --- | --- |
| **CSF test** |  |  |  |  |  |  |  |  |
| Intracranial pressure (mmH_2_O) | 80-180 |  | 150 |  |  | 70 | 70 |  |
| Color | colorless |  | yellowish |  |  | colorless | colorless | colorless |
| Pandy test | negative |  | positive |  |  | positive | positive | positive |
| total leukocytes count  (10^6^/L) | 0-8 |  | 97 |  |  | 8 | 10 | 10 |
| mononuclear leukocytes  percentage |  |  | 70% |  |  | 90% | 90% | 60% |
| polymorphonuclear  leukocytes percentage |  |  | 30% |  |  | 10% | 10% | 40% |
| glucose (mmol/L) | 2.50-4.50 |  | 1.79 |  |  | 4.21 | 2.84 | 3.16 |
| chloride (mmol/L) | 120.0-132.0 |  | 115.6 |  |  | 116.8 | 121.6 | 128.4 |
| protein (mg/L) | 150.0-450.0- |  | 2492.6 |  |  | 1863.3 | 1638.9 | 758.6 |

CSF, cerebrospinal fluid.
